# Accuracy comparison of single- and double-sleeve endodontic guides for fiber post removal

**DOI:** 10.1186/s12903-024-04283-w

**Published:** 2024-04-27

**Authors:** Omid Dianat, Mandana Naseri, Yaser Safi, Ali Modaberi, Nazanin Zargar, Ove A. Peters, Mehran Farajollahi

**Affiliations:** 1 Division of Endodontics, Department of Advanced Oral Sciences and Therapeutics, School of Dentistry, University of Maryland, Baltimore, MD USA; 2 Department of Endodontics, School of Dentistry, Shahid Beheshti University of Medical Sciences, Tehran, Iran; 3 Department of Oral and Maxillofacial Radiology, School of Dentistry, Shahid Beheshti University of Medical Sciences, Tehran, Iran; 4 School of Dentistry, The University of Queensland, Herston, QLD Australia; 5 Iranian Center for Endodontic Research, Research Institute of Dental Sciences, School of Dentistry, Shahid Beheshti University of Medical Sciences, Tehran, Iran

**Keywords:** Endodontics, Root canal therapy, Cone-beam computed tomography, Intracanal posts, Guided endodontics

## Abstract

**Background:**

This study aimed to assess the accuracy of two different endodontic guides for fiber post removal.

**Methods:**

In this in vitro study, 54 maxillary canine fiber posts were mounted on 36 maxillary stone casts; 18 teeth were mounted unilaterally, and 36 teeth were mounted bilaterally. Static endodontic guides were fabricated according to baseline cone-beam computed tomography (CBCT) and intraoral optical scans using Blue Sky software. In the single-sleeve endodontic guides group (SSG), two anterior and two posterior teeth were included in a 5-unit guide. In the double-sleeve endodontic guides group (DSG) group, the guide was passed through the midline to include both canine teeth and extended by 2 teeth posterior to the canine teeth bilaterally (a 10-unit guide). After drilling, postoperative CBCT scans were taken and superimposed on the virtually designed path, and the maximum coronal deviation (MCD) at the marginal entry point of the tooth, maximum apical deviation (MAD) at 10 mm apical to the tooth margin, and maximum angular deflection (MAnD) of the drill were calculated.

**Results:**

The mean MCD, MAD, and MAnD were 0.34 mm, 0.6 mm, and 2.32 degrees, respectively, in the SSG and 0.31 mm, 0.7 mm, and 2.37 degrees, respectively, in the DSG. The two groups were not significantly different from each other in terms of MCD (*P* = 0.573), MAD (*P* = 0.290), or MAnD (*P* = 0.896).

**Conclusions:**

The accuracies of the two techniques, the extended double sleeve guide and the single sleeve guide, were comparable and thus DSG may be used for removal of fiber posts in adjacent or distant teeth.

## Background

The main goal of primary nonsurgical endodontic treatment is to prevent or treat apical periodontitis [1]. Periapical lesions may develop again after poor-quality primary endodontic treatment or via coronal leakage. Root canal retreatment should be performed for such cases, if feasible [2]. Elimination of barriers such as intracanal posts against accessing canals is among the challenges encountered in root canal retreatment [3, 4].

Several parameters need to be considered when selecting an intracanal post, such as the modulus of elasticity of the post compared to that of dentin, the stress distribution along the longitudinal root axis, and the risk of root fracture [5, 6]. Other parameters, such as aesthetics, tooth type/location, and parafunctional habits, should also be taken into account [7]. Cast posts may cause root fracture due to stress accumulation. Thus, the application of fiber posts has gained increasing attention since they absorb and transfer stresses to the residual tooth structure more uniformly because they have a modulus of elasticity close to that of dentin [8]. Optimized aesthetics [9], biocompatibility, translucency, direct use in the clinical setting, and decreased number of required treatment sessions of these materials further contributed to their increasing use [10].

Endodontic retreatment requires intracanal post removal from the root canal system. Thus, a standard technique for post removal would be highly useful [11]. Retrieval of fiber posts is often difficult due to the application of novel bonding techniques for luting [12, 13]. Several different techniques have been suggested for fiber post removal from canals, such as the use of diamond burs, Gates Glidden drills, Largo drills, Peeso reamers, special fiber post retrieval kits, ultrasonic instruments, and Er: YAG lasers along with a microscope. Each technique is indicated for use at a certain time, and each technique preserves a different amount of residual tooth structure [12, 14–17]. However, all the above-mentioned techniques are time-consuming and can be challenging. Additionally, they can mechanically injure the root canal and adversely affect the long-term prognosis of treatment [18]. Thus, post removal should be performed atraumatically with maximum preservation of the residual tooth structure to guarantee long-term clinical service [19].

Recently, an alternative technique has been introduced for fiber post removal by using a static guide and tomographic and optical images [20]. Studies on the accuracy of endodontic guides for accessing root canals have reported satisfactory results [21, 22]. Endodontic guides are indicated for access cavity preparation in teeth with calcified canals, for cases of apicoectomy, for fiber post removal from the root canals, and for endodontic treatment of teeth with complex anatomy [23].

At present, endodontic guides are divided into two groups: dynamic and static. Dynamic guides are real-time intraoperative navigation systems that monitor the correct implementation of a predesigned treatment plan by using cameras [24]. Static guides are three-dimensionally printed templates made of resin. They are placed over the teeth to guide the drill. To fabricate a static guide, a cone-beam computed tomography (CBCT) scan and optical impression of the area are required [25]. The advantages of endodontic static guides include faster procedures and greater accuracy and safety than conventional endodontic methods, regardless of the clinician’s experience. The disadvantages of these methods include limited application to straight canals only and time-consuming design and printing [23]. The application of a guide for fiber post removal can enhance this process, increase the safety of the procedure, and aid in greater preservation of the root structure [26].

The accuracy of double-sleeve endodontic guides (DSGs) has not been previously evaluated for fiber post removal from more than one tooth. Thus, this study aimed to assess the accuracy of single-sleeve (SSG; with one single guiding hole for one single tooth) versus double-sleeve (DSG: extended design with two guiding holes for two teeth) endodontic guides for fiber post removal.

## Methods

This in vitro experimental study was conducted on 54 single-rooted maxillary canine teeth extracted for reasons not related to this study, such as poor periodontal prognosis. The study protocol was approved by the ethics committee of the university (IR.SBMU.DRC.REC.1401.060).

### Sample size

The sample size for this study was calculated to be 18 in each group assuming α = 0.05, β = 0.2, a study power of 80%, mean values of 1.2 and 1.9, and standard deviation values of 0.6 and 0.8 for global apical deviation based on data reported in a previous study [27]. In the SSG group, canine teeth were mounted unilaterally (*n* = 18), while in the DSG group, two canine teeth were mounted bilaterally in each cast (*n* = 36). A total of 36 casts and three-dimensionally printed templates were evaluated.

### Eligibility criteria

The inclusion criteria were a mean length of 25–27 mm, completely formed roots, having a single canal, small/no caries, and no/minimal restoration.

### Specimen preparation

For the purpose of standardization, teeth were decoronated by a disc such that the root length was standardized at 20 mm.

After access cavity preparation, a #15 K-file (Mani, Tochigi-Ken, Japan) was introduced into the canal to ensure patency and to determine the working length. The root canals were then instrumented with ProTaper Gold (Dentsply Sirona, Ballaigues, Switzerland) using the single-length technique. All teeth were instrumented to F3 final size and irrigated with 2 mL 5.25% sodium hypochlorite between the files using a 30-gauge side-vented needle with a safety tip. The total volume of irrigation with 5.25% sodium hypochlorite at the end of the instrumentation was 10 mL for each canal. After completion of the root canal preparation, 5 mL 17% EDTA was applied to the canals for 1 min, after which the specimens were subsequently rinsed with saline. After the root canals were dried at paper points, the canals were obturated via the single cone technique using the corresponding ProTaper gutta-percha points and AH26 sealer (Dentsply Sirona). The specimens were wrapped in sterile gauze and stored at 100% humidity (to remain hydrated) for 1 week to allow the sealer to set.

### Post space preparation

The post space was prepared with a #4 Peeso reamer (Mani, Tochigi – Ken, Japan) 1.3 mm in diameter and 11 mm in length from the tooth surface. A #2 cylindrical glass fiber post (Nordin Glassix, Chailly, Switzerland) with a round end and a 1.2 mm diameter was inserted into the canal, and a fissure bur was used to shorten the fiber post 2 mm below the tooth margin for placement of the final restoration. The fiber post was then cemented in the canal by using self-adhesive resin cement (DentKist SuperCem, Gunpo-si, South Korea). Finally, a dual-cure composite (Tokuyama, Tokyo, Japan) with a 2 mm thickness was applied over the fiber post.

### Mounting

**Fig. 1:**
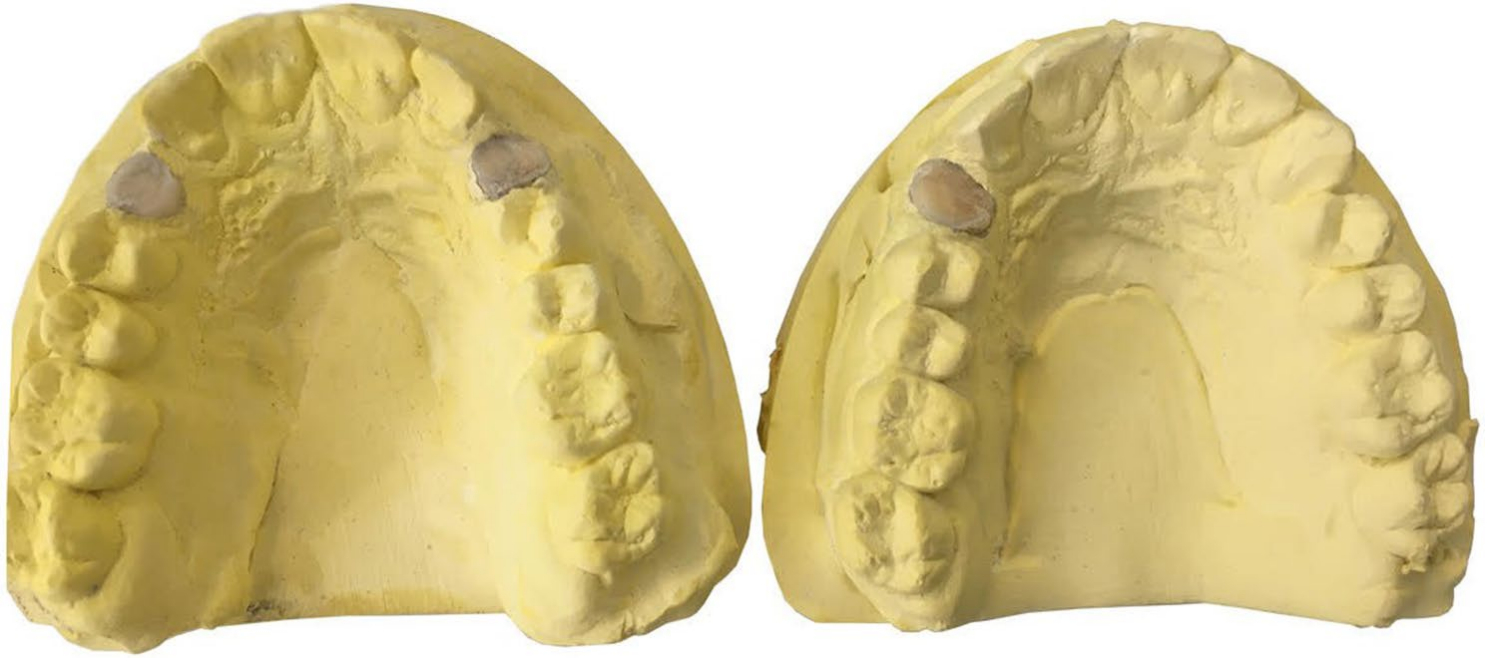
(Right) SSG group stone cast. (Left) DSG group stone cast

The teeth were mounted in 36 stone casts. In the SSG group, canine teeth were mounted unilaterally (*n* = 18; 9 on each side, R or L) to mitigate any potential left-hand or right-hand bias, while in the DSG group, two canine teeth were mounted bilaterally in each cast. To fix the teeth in the casts, a putty-wash impression was made from the maxillary dental arch of a resin model, and the extracted canine teeth were mounted at the site of canine teeth in the impression by using dental wax. The impressions were then poured with type 3 dental stone. The teeth in the impression were embedded in dental wax from their cusp tip to their cementoenamel junction (CEJ). Thus, the coronal parts above the CEJ remained exposed, while the areas below the CEJ were embedded and fixed in dental stone (Fig. 1).

### Designing and application of guides

All casts were subjected to CBCT with a NewTom VGi CBCT scanner (NewTom, Verona, Italy) with the following parameters: 110 kVp, 11.21 mA current, 60 × 60 mm field of view, and 0.15 mm voxel size. The scans were saved in DICOM format. One cast from the SSG group and one from the DSG group were visualized via CBCT, and the images were separated from each other by a cotton roll. After CBCT, all casts were fully scanned by an intraoral scanner (CS 3600 scanner; Carestream, Atlanta, USA), and the files were saved in STL format.

**Fig. 2:**
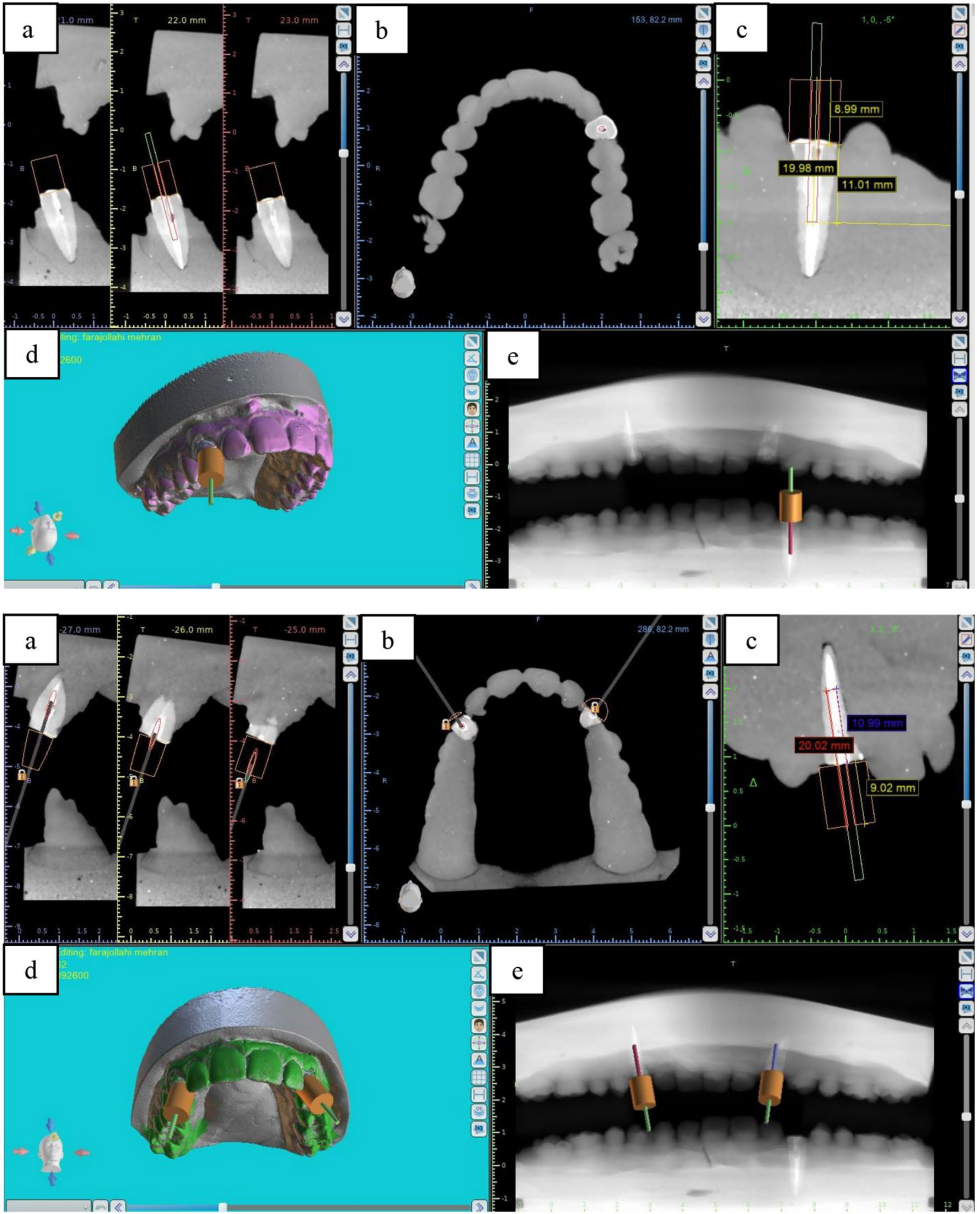
(Up) Guide design for the SSG group. (Down) Guide design for the DSG group. (**a**) Coronal, (**b**) axial, (**c**) sagittal, (**d**) three-dimensional, and (**e**) panoramic views

**Fig. 3:**
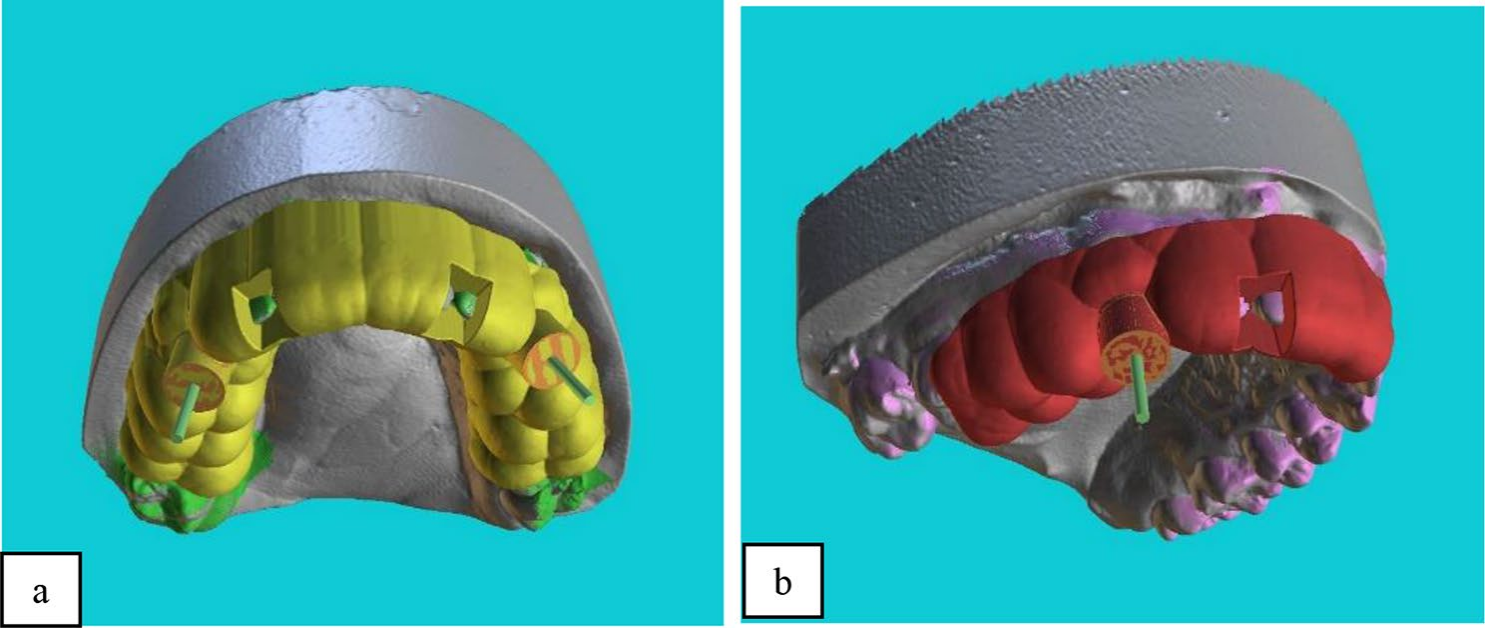
Dental coverage, location of the metal sleeve, verification windows of the guide, and location of the drill entry in the DSG (**a**) and SSG (**b**) groups

Both the CBCT and intraoral optical scan files were transferred to surgical planning software (Blue Sky Plan 4; Blue Sky Bio, LLC; Grayslake, IL) for automatic superimposition. An expert operator also supervised the process to ensure accurate superimposition of the two files. After superimposition of the files, the guide was designed according to the location of the fiber post such that the drill would have no contact with the root dentin and would only drill the fiber post along its path. The drilling path was terminated when the end of the fiber post was reached (11 mm from the tooth surface), and gutta-percha root filling was initiated (Fig. 2). With respect to tooth coverage by the guide, in the SSG group, two anterior and two posterior teeth were included in the guide design (a 5-unit guide). In the DSG, the guide was passed through the midline, which included both canines bilaterally and two teeth posterior to the canine teeth on each side (a 10-unit guide) (Fig. 3). The location of the metal sleeve in the resin template was also determined. The stainless-steel metal sleeve (Straumann T-sleeve, Basel, Switzerland) used in this study had a 2.5 mm external diameter, 1.35 mm internal diameter, and 7.5 mm length [28]. Additionally, to stop the drill at a length of 20 mm, a 1.5 mm space was considered between the metal sleeve and the tooth surface.

**Fig. 4:**
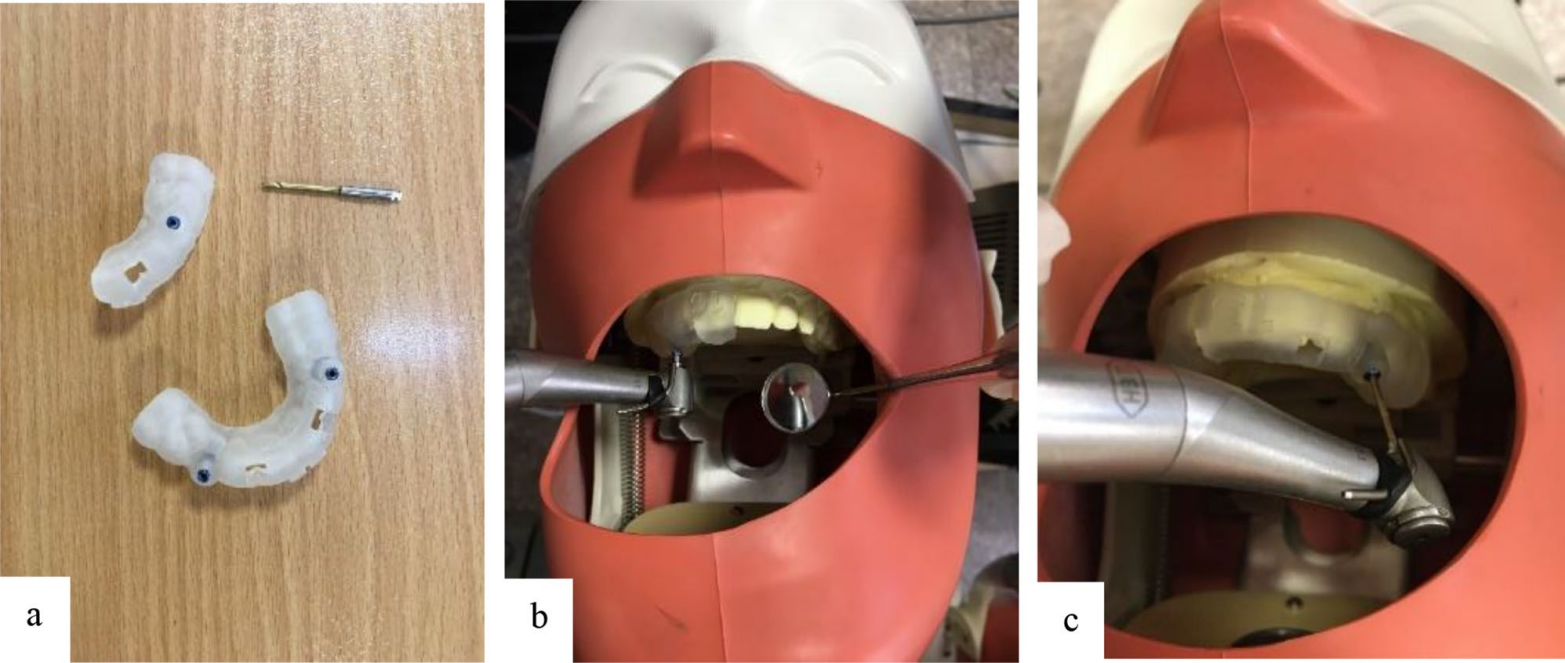
(**a**, **b**, **c**) Drilling process; SSG and DSG along with the drill

The design was then printed with a 3D printer (Anycubic, Shenzhen, China) using photopolymerized biocompatible polymer resin (PowerResins, Singapore) with a 3 mm thickness. The metal sleeve was placed mechanically (frictionally) in the created hole due to the presence of threads in the sleeve. The templates were tested in their respective casts to ensure their correct seating, retention, and stability. Additionally, verification windows were considered in resin in template design to ensure complete seating of the guide. The casts were placed in a phantom head and fixed by an incorporated magnet. The correctness of the guide on the cast was evaluated again by the verification window (Fig. 4).

A Straumann drill (Straumann Guided Surgery, Basel, Switzerland) 20 mm in length and 1.3 mm in diameter was used in combination with an implant motor (NSK, Nakanishi, Japan) operating at 1200 rpm with 25 N/cm torque for drilling of the fiber post to reach the gutta-percha [28]. Drilling of each tooth was performed in 3 steps. After each step, the guide was removed, and the drill debris, sleeve, and tooth were rinsed with saline. The entire drill length (20 mm) was entered into the metal sleeve such that 9 mm of the drill was involved with the sleeve and the distance between the sleeve and tooth surface, and the apical 11 mm of the drill was in the canal for fiber post removal. After completion of drilling, 17% EDTA and saline were used for the final rinse of debris. The entire drilling process was performed by a senior postgraduate student in endodontics (Fig. 4).

**Fig. 5:**
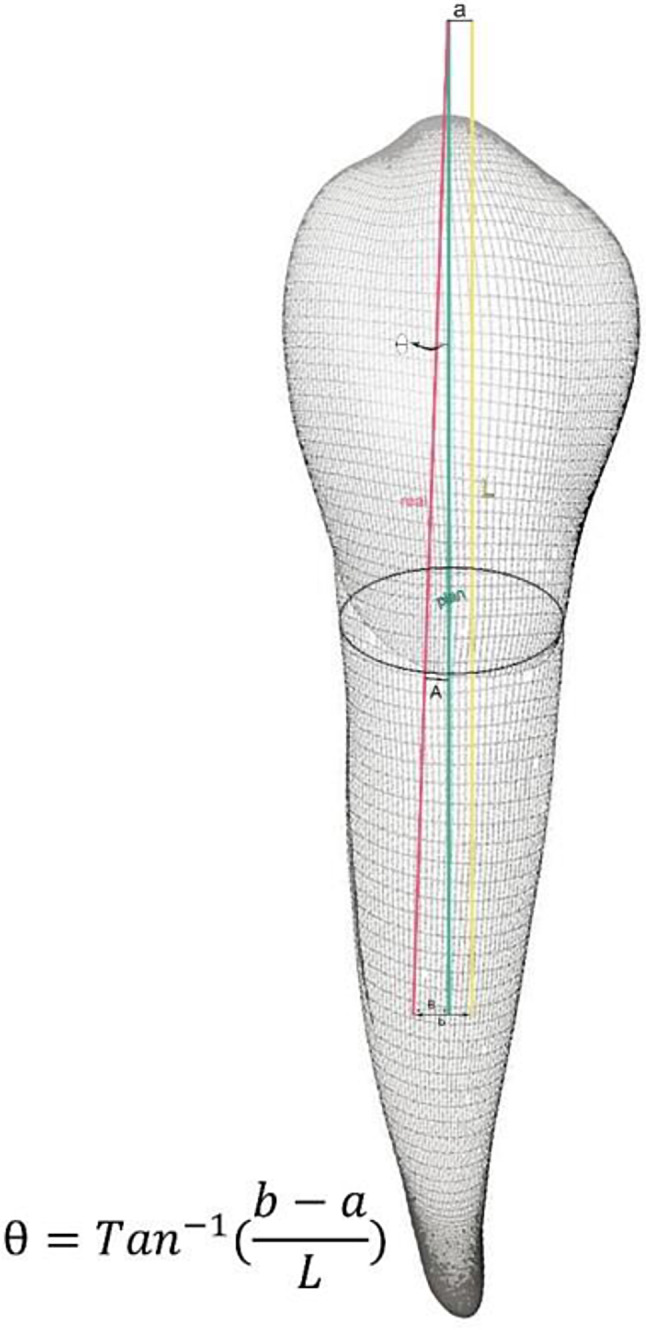
A schematic view of the maximum coronal deviation (**A**), maximum apical deviation (**B**), and maximum angular deflection (Ɵ) is shown

**Fig. 6:**
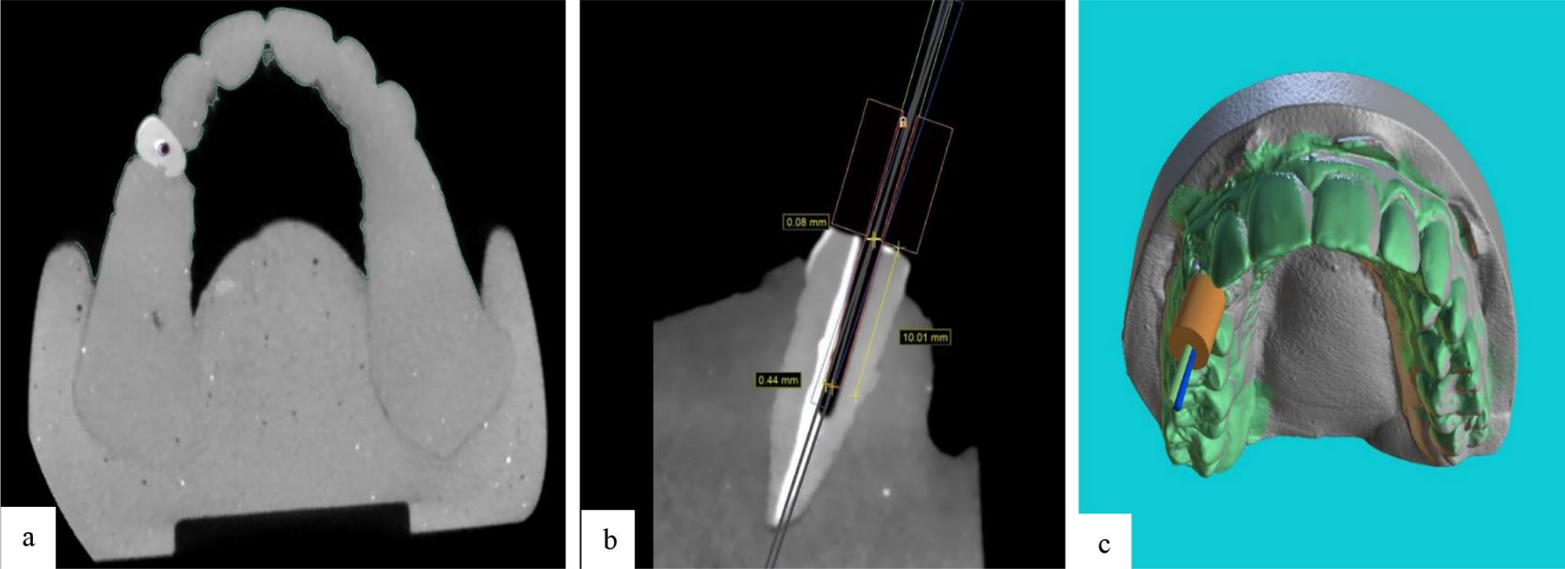
(**a**) Superimposition of postoperative CBCT scans on the virtually designed drill path in the SSG group (the axial view (red circle) indicates the virtually designed path, and the blue circle indicates the actual drill path). (**b**) Calculation of the maximum coronal deviation (at 0 mm) and maximum apical deviation at 10 mm apical to the tooth margin in the SSG group (the blue rectangle indicates the virtually designed path, and the red rectangle indicates the actual drill path). (**c**) Superimposition of postoperative CBCT scan on the virtually designed drill path in the SSG group (3D view - green cylinder indicates the virtually designed path, and blue cylinder indicates the actual drill path)

**Fig. 7:**
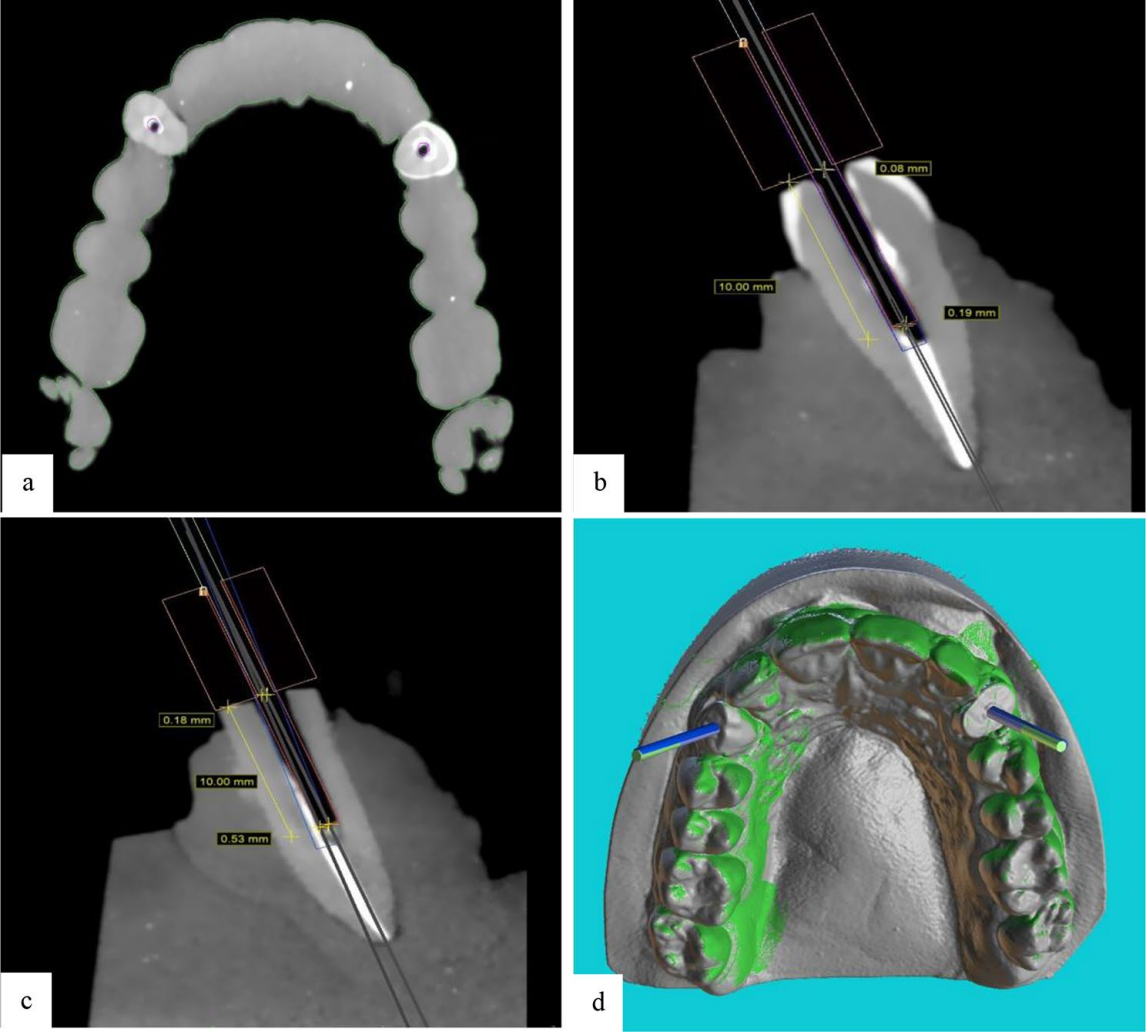
(**a**) Superimposition of postoperative CBCT scans on the virtually designed drill path in the DSG group (the axial view (red circle) indicates the virtually designed path, and the blue circle indicates the actual drill path). (**b**, **c**) Calculations of the maximum coronal deviation (at 0 mm) and maximum apical deviation at 10 mm apical to the tooth margin in the DSG for the right and left canines, respectively (the blue rectangle indicates the virtually designed path, and the red rectangle indicates the actual drill path). (**d**) Superimposition of postoperative CBCT scans on the virtually designed drill path in the DSG group (3D view: the green cylinder indicates the virtually designed path, and the blue cylinder indicates the actual drill path)

After completion of drilling, all casts in both groups underwent CBCT with the same parameters as explained for the preoperative CBCT. The postoperative CBCT scans were subsequently transferred to Blue Sky software (Blue Sky Plan 4; Blue Sky Bio, LLC, Grayslake, IL). The drill paths on the postoperative CBCT scans were superimposed on the virtually designed drill path, and the maximum coronal deviation (MCD) at the marginal entry point of the tooth in millimeters (mm), the maximum apical deviation (MAD) at 10 mm apical to the tooth margin in millimeters (mm), and the maximum angular deflection (MAnD) from the designed path in degrees were calculated and recorded (Figs. 5, 6 and 7).

### Statistical analysis

The Shapiro‒Wilk test confirmed the normality of the data distribution, while Levene’s test confirmed the homogeneity of variance (*P* > 0.05). Thus, an independent t test was applied to compare the means of the dependent variables between the two groups (SSG and DSG). All the statistical analyses were carried out using SPSS version 25 (SPSS, Inc., IL, USA) at the 0.05 level of significance.

## Results

In the SSG group, the means of the MCD, MAD, and MAnD were 0.34 mm, 0.6 mm, and 2.32 degrees, respectively. Furthermore, in the DSG group, the means of the MCD, MAD, and MAnD were 0.31 mm, 0.7 mm, and 2.37 degrees, respectively. Table 1 shows the MCD, MAD, and MAnD values of the two groups. Perforation did not occur in any tooth. In both groups, the MAD of the drill was greater than its MCD. According to the results of an independent t test (Table 2), the two groups were not significantly different from each other in terms of MCD (*P* = 0.573), MAD (*P* = 0.290), or MAnD (*P* = 0.896). The mean differences between the two groups in MCD, MAD, and MAnD were 0.03 mm, 0.1 mm, and 0.05 degrees, respectively.

**Table 1:** MCD, MAD, and MAnD in the two groups

		N	Mean	Std. Deviation	95% Confidence Interval for Mean		Minimum	Maximum
					Lower Bound	Upper Bound		
Maximum coronal deviation	SSG	18	0.3472	0.20390	0.2458	0.4486	0.07	0.77
	DSG	36	0.3169	0.17475	0.2578	0.3761	0.02	0.67
Maximum apical deviation	SSG	18	0.6022	0.35788	0.4243	0.7802	0.04	1.17
	DSG	36	0.7094	0.34213	0.5937	0.8252	0.03	1.33
Maximum angular deflection	SSG	18	2.3278	1.42149	1.6209	3.0347	0.17	5.31
	DSG	36	2.3792	1.32358	1.9313	2.8270	0.29	5.43

SSG Single-sleeve guide; DSG Double-sleeve guide

**Table 2:** Comparison of the variables between the two groups by an independent t test

		Levene’s Test for Equality of Variances		t test for Equality of Means			
		F	Sig.	t	df	Sig. (2-tailed)	Mean Difference
Maximum coronal deviation	Equal variances assumed	0.386	0.537	0.568	52	0.573	0.03028
Maximum apical deviation	Equal variances assumed	0.340	0.562	−1.069	52	0.290	− 0.10722
Maximum angular deflection	Equal variances assumed	0.000	0.986	− 0.131	52	0.896	− 0.05139

## Discussion

This study compared the accuracy of the SSG and DSG methods for removing fiber posts. Maxillary canine teeth were used in the present study since they are located at the point of maximum bending of the maxillary arch. The results showed no significant difference in MCD, MAD, or MAnD between the two groups. Thus, in the case of requiring fiber post removal from two teeth, the static endodontic guide can be extended to adjacent teeth or the other quadrant of the dental arch with no adverse effects on its accuracy. By doing so, the need for the fabrication of a separate guide for adjacent teeth or the other quadrant would be eliminated.

Previous in vitro studies have shown the optimal efficacy and accuracy of endodontic static guides for fiber post removal [29, 30]. The MCD, MAD, and MAnD values in the present study were comparable to the values reported in the literature. Despite some degree of deviation, continuation of the canal path and access to the gutta-percha filling were possible for all teeth, and no perforation occurred. According to Buchgreitz et al. [22], the accuracy of the guide should be considered acceptable when the canal path is preserved and when the canal is successfully prepared. Thus, the guides fabricated in the present study were successful and can be used as an efficient and predictable tool for fiber post removal.

In the present study, dental coverage was extended in the DSG group to increase the stability and accuracy of the guide. Nonetheless, no significant difference was found in the SSG group, which had minimal dental coverage (two anterior and two posterior teeth). Thus, in the case of requiring fiber post removal from only one tooth, minimum dental coverage can provide optimal stability comparable with the use of an extended guide.

Tooth cracking is a possible problem associated with the use of endodontic guides due to pressure applied to the drill tip during the drilling process [16, 28, 31]. This pressure can generate heat and damage the periodontal ligament and the supporting alveolar bone [32]. Heat generation may be intensified if the coolant does not reach the drill tip [33]. In the present study, a crack was noted in one tooth after the drilling process, which could have been caused by several factors, such as the implant motor torque during the drilling process and the pressure applied to the drill tip, as well as the mounting of the teeth in a stone cast and tooth dehydration. To prevent locking of the drill in the sleeve and subsequent friction and heat generation, a small space is considered between the drill and sleeve, which can decrease the accuracy of endodontic static guides. In the present study, the difference in diameter between the drill and sleeve was 0.05 mm, which could cause off-axial movements of the drill and subsequent reductions in the accuracy of the guide. As mentioned earlier, the drill used in this study was 20 mm long and 1.3 mm in diameter along its entire length; thus, the drill was parallel to the sleeve during use in the endodontic guide, and a 0.05 mm space was maintained with the metal sleeve in the entire path. Using convergent or divergent drills can affect this space and subsequently lower the accuracy of endodontic guides [34].

The problems related to poor vision and access of irrigant to the drill tip in the use of endodontic guides were resolved by the introduction of sleeveless endodontic guides that guide the handpiece instead of the drill. In an in vitro study, Mo et al. [35] evaluated the accuracy of sleeveless endodontic guides compared with the free-handed technique for fiber post removal and reported greater accuracy of the sleeveless endodontic guide. The values reported in their study were close to those obtained in the present study.

In the present study, the drilling length required to reach the gutta-percha was 11 mm. For teeth with greater deviation, the drill did not reach 11 mm in length. According to Connert et al. [36], differences in the hardness of different materials used for tooth restoration can affect the drill path such that deviation toward the tooth structure can result in greater pressure applied by the operator to reach the desired length. It appears that not reaching the desired length in teeth with greater deviation in the present study was due to drill deviation and contact with the hard root dentin. All the teeth were drilled to a length of 10 mm. Thus, the MAD was calculated at 10 mm apical to the tooth margin.

Considering the present results and the available clinical literature supporting the successful use of endodontic static guides for fiber post removal [29, 30, 34, 37], clinicians should be well aware of the parameters that can decrease the accuracy of such guides. In a case report by Fonseca Tavares et al. [38], an endodontic guide could not be successfully used to access a calcified root canal path in the central incisor tooth, which led to root perforation. The observed low accuracy of the guide in their study was attributed to the absence of fixing screws for guide stabilization and the manual execution of mesh merging software. Additionally, it should be noted that the present study and other in vitro investigations on the accuracy of endodontic guides for fiber post removal [29, 30, 35] have been conducted under ideal conditions, which are different from the clinical setting. The stability of the guide in the clinical setting can be challenging considering poor vision, the presence of the tongue, and the applied muscle forces. Moreover, limited mouth opening and limited interocclusal distance can complicate proper use of static guides in the posterior areas due to the space occupied by the sleeve [35]. Furthermore, in the present study, a postoperative CBCT scan was obtained immediately after drilling, which cannot be routinely performed in the clinical setting due to concerns about radiation exposure. Torres et al. [39] performed an intraoral optical scan immediately after drilling and without removing the drill from the drilled space. The authors superimposed the postoperative optical scan on the preoperative CBCT scan and preoperative optical scan to assess the accuracy of the guide. They concluded that postoperative optical scans can be beneficial for assessing the accuracy of endodontic guides as a safe alternative to CBCT. Additionally, optical scanning does not require moving the patient and can be performed in a shorter time than can CBCT [39].

According to Fachin et al. [30], clinicians should have sufficient knowledge about the parameters that can decrease the accuracy of the guide and acquire the necessary clinical skills through in vitro practice due to the high technical sensitivity of the procedure to maximize the chance of a good prognosis and prevent iatrogenic errors in using an endodontic guide.

In the present study, all teeth were decapitated to eliminate the possible confounding effect of anatomical variations in tooth crowns, and the root length was standardized to 20 mm, which was considered good.

The in vitro design of the present study was a limitation that limits the generalizability of the findings to the clinical setting. Another limitation of the experimental design lies in the need to decoronate the long canine teeth selected for necessary standardization and proper placement of fiber post inside the canal. This resulted in shortened distances and may reduce the stringency of the conditions for drill guide accuracy. Future studies are recommended to compare the accuracy of DSGs and SSGs for identifying calcified canals. Additionally, the accuracy of different drill holes and printers for fiber post removal should be compared to achieve a standard clinical protocol.

## Conclusion

The accuracy of the DSG (extended to the other quadrant for fiber post removal from two teeth) and the SSG (designed for one quadrant for fiber post removal from one tooth) were comparable and not significantly different. Thus, DSG may be used for removal of fiber posts from adjacent teeth or the other quadrant of the dental arch, with no concern regarding a compromise in accuracy. Furthermore, increasing the dental coverage to enhance stability had no significant effect on the accuracy of the endodontic guide, and the minimum coverage for fiber post removal from one single tooth had a comparable accuracy to that of the extended form.

## Data Availability

The datasets used and analyzed during the current study are available from the corresponding author on reasonable request.

## Abbreviations

SSG: Single-sleeve endodontic guide
DSG: Double-sleeve endodontics guide
MCD: Maximum coronal deviation
MAD: Maximum apical deviation
MAnD: Maximum angular deflection
CBCT: Cone-beam computed tomography
CEJ: Cementoenamel junction
EDTA: Ethylenediaminetetraacetic acid
3D: Three dimensional
DICOM: Digital imaging and Communication in Medicine
STL: Standard Tessellation Language

## References

[1] Friedman S (2002). Prognosis of initial endodontic therapy. Endod Top.

[2] Ruddle CJ (2004). Nonsurgical retreatment. J Endod.

[3] Curtis DM, VanderWeele RA, Ray JJ, Wealleans JA (2018). Clinician-centered outcomes Assessment of Retreatment and Endodontic Microsurgery Using Cone-Beam Computed Tomographic Volumetric Analysis. J Endod.

[4] Dickie J, McCrosson J (2014). Post removal techniques part 1. Dent Update.

[5] Coelho CS, Biffi JC, Silva GR, Abrahão A, Campos RE, Soares CJ (2009). Finite element analysis of weakened roots restored with composite resin and posts. Dent Mater J.

[6] Maroulakos G, Nagy WW, Kontogiorgos ED (2015). Fracture resistance of compromised endodontically treated teeth restored with bonded post and cores: an in vitro study. J Prosthet Dent.

[7] Naumann M, Blankenstein F, Kiessling S, Dietrich T (2005). Risk factors for failure of glass fiber-reinforced composite post restorations: a prospective observational clinical study. Eur J Oral Sci.

[8] Ferrari M, Vichi A, García-Godoy F (2000). Clinical evaluation of fiber-reinforced epoxy resin posts and cast post and cores. Am J Dent.

[9] Lamichhane A, Xu C, Zhang FQ (2014). Dental fiber-post resin base material: a review. J Adv Prosthodont.

[10] Niu D, Xie J, Liu C, Ni S, Liu H (2021). The influence of different treatments on fiber post and root canal to bond strength of fiber post. J Adhes Sci.

[11] Anderson GC, Perdigão J, Hodges JS, Bowles WR (2007). Efficiency and effectiveness of fiber post removal using 3 techniques. Quintessence Int.

[12] Lindemann M, Yaman P, Dennison JB, Herrero AA (2005). Comparison of the efficiency and effectiveness of various techniques for removal of fiber posts. J Endod.

[13] Cormier CJ, Burns DR, Moon P (2001). In vitro comparison of the fracture resistance and failure mode of fiber, ceramic, and conventional post systems at various stages of restoration. J Prosthodont.

[14] Deeb JG, Grzech-Leśniak K, Weaver C, Matys J, Bencharit S (2019). Retrieval of Glass Fiber Post using Er:YAG Laser and Conventional Endodontic Ultrasonic Method: an in Vitro Study. J Prosthodont.

[15] Abe FC, Bueno CE, De Martin AS, Davini F, Cunha RS (2014). Efficiency and effectiveness evaluation of three glass fiber post removal techniques using dental structure wear assessment method. Indian J Dent Res.

[16] Çapar İD, Uysal B, Ok E, Arslan H (2015). Effect of the size of the apical enlargement with rotary instruments, single-cone filling, post space preparation with drills, fiber post removal, and root canal filling removal on apical crack initiation and propagation. J Endod.

[17] Gesi A, Magnolfi S, Goracci C, Ferrari M (2003). Comparison of two techniques for removing fiber posts. J Endod.

[18] Haupt F, Pfitzner J, Hülsmann M (2018). A comparative in vitro study of different techniques for removal of fibre posts from root canals. Aust Endod J.

[19] Arukaslan G, Aydemir S (2019). Comparison of the efficacies of two different fiber post-removal systems: a micro-computed tomography study. Microsc Res Tech.

[20] Maia LM, Bambirra Júnior W, Toubes KM, Moreira Júnior G, de Carvalho Machado V, Parpinelli BC (2022). Endodontic guide for the conservative removal of a fiber-reinforced composite resin post. J Prosthet Dent.

[21] Zehnder MS, Connert T, Weiger R, Krastl G, Kühl S (2016). Guided endodontics: accuracy of a novel method for guided access cavity preparation and root canal location. Int Endod J.

[22] Buchgreitz J, Buchgreitz M, Mortensen D, Bjørndal L (2016). Guided access cavity preparation using cone-beam computed tomography and optical surface scans - an ex vivo study. Int Endod J.

[23] Kulinkovych-Levchuk K, Pecci-Lloret MP, Castelo-Baz P, Pecci-Lloret MR, Oñate-Sánchez RE. Guided endodontics: a Literature Review. Int J Environ Res Public Health. 2022;19(21). 10.3390/ijerph192113900 PMC9657991 36360780

[24] Zubizarreta-Macho Á, Muñoz AP, Deglow ER, Agustín-Panadero R, Álvarez JM. Accuracy of computer-aided dynamic Navigation compared to computer-aided Static Procedure for Endodontic Access cavities: an in Vitro Study. J Clin Med. 2020;9(1). 10.3390/jcm9010129 PMC7019931 31906598

[25] Kim BN, Son SA, Park JK (2021). Endodontic retreatment of a calcified anterior tooth using a 3D-printed endodontic guide. Int J Comput Dent.

[26] Maia LM, Moreira Júnior G, Albuquerque RC, de Carvalho Machado V, da Silva N, Hauss DD (2019). Three-dimensional endodontic guide for adhesive fiber post removal: a dental technique. J Prosthet Dent.

[27] Janabi A, Tordik PA, Griffin IL, Mostoufi B, Price JB, Chand P (2021). Accuracy and efficiency of 3-dimensional dynamic Navigation System for removal of Fiber Post from Root Canal-treated Teeth. J Endod.

[28] Krastl G, Zehnder MS, Connert T, Weiger R, Kühl S (2016). Guided endodontics: a novel treatment approach for teeth with pulp canal calcification and apical pathology. Dent Traumatol.

[29] Perez C, Sayeh A, Etienne O, Gros CI, Mark A, Couvrechel C (2021). Microguided endodontics: accuracy evaluation for access through intraroot fibre-post. Aust Endod J.

[30] Fachin GF, Dinato TR, Prates FB, Connert T, Pelegrine RA, Bueno CES (2023). Guided Access through Ceramic crowns with Fiberglass Post removal in Lower molars: an in Vitro Study. Appl Sci.

[31] Fonseca Tavares WL, Diniz Viana AC, de Carvalho Machado V, Feitosa Henriques LC, Ribeiro Sobrinho AP (2018). Guided Endodontic Access of Calcified Anterior Teeth. J Endod.

[32] Saunders EM, Saunders WP (1989). The heat generated on the external root surface during post space preparation. Int Endod J.

[33] Kwon SJ, Park YJ, Jun SH, Ahn JS, Lee IB, Cho BH (2013). Thermal irritation of teeth during dental treatment procedures. Restor Dent Endod.

[34] Farajollahi M, Dianat O, Gholami S, Saber Tahan S. Application of an Endodontic Static Guide in Fiber Post Removal from a Compromised Tooth. Case Rep Dent. 2023;2023. 10.1155/2023/7982368 PMC10516697 37745692

[35] Mo S, Xu Y, Zhang L, Cao Y, Zhou Y, Xu X (2023). Accuracy of a 3D printed sleeveless guide system used for fiber post removal: an in vitro study. J Dent.

[36] Connert T, Krug R, Eggmann F, Emsermann I, ElAyouti A, Weiger R (2019). Guided endodontics versus Conventional Access Cavity Preparation: a comparative study on substance loss using 3-dimensional-printed Teeth. J Endod.

[37] Alfadda A, Alfadley A, Jamleh A (2022). Fiber post removal using a conservative fully guided Approach: a Dental technique. Case Rep Dent.

[38] Fonseca Tavares WL, de Oliveira Murta Pedrosa N, Moreira RA, Braga T, de Carvalho Machado V, Ribeiro Sobrinho AP (2022). Limitations and Management of Static-guided endodontics failure. J Endod.

[39] Torres A, Dierickx M, Coucke W, Pedano MS, Lambrechts P, Jacobs R (2023). Ex-vivo and in-vivo validation of a novel measuring protocol for guided endodontics. J Dent.

